# How the proposal for a new regulation for new genomic techniques affects the European Union’s food system sustainability objectives

**DOI:** 10.1038/s44264-026-00154-9

**Published:** 2026-06-02

**Authors:** Maximilian Kardung, Samuel Ahado, Yasmine Ambrogio, Lukáš Čechura, Kutay Cingiz, Dennis Eriksson, João Godinho, Yan Jin, Dimitrios G Karpouzas, Stelios Kartakis, Zuzana Smeets Kristkova, Justus Wesseler

**Affiliations:** 1 Agricultural Economics and Rural Policy Group, Wageningen University, Wageningen, The Netherlands; 2 Deloitte Germany, Berlin, Germany; 3 Technology Centre Prague, Department of Strategic Studies (STRAST), Prague, Czech Republic; 4 Chair of Food Law, University of Bayreuth, Kulmbach, Germany; 5 Czech University of Life Sciences, Prague, Czech Republic; 6 Swedish University of Agricultural Sciences, Alnarp, Sweden; 7 Wageningen Economic and Social Research, The Hague, The Netherlands; 8 Department of Biochemistry and Biotechnology, University of Thessaly, Larissa, Greece

**Keywords:** Ecology, Ecology, Environmental sciences, Plant sciences

## Abstract

The European Commission has proposed a regulation for plants developed through new genomic techniques to enhance the sustainability of the food system. The proposal features a two-tiered pathway exempting ‘Category 1’ plants from full genetically modified organisms legislation. This approach could positively impact low-risk pesticide usage and invasive species management. The success of these techniques depends on alignment with effective farm management strategies, addressing regulatory challenges, and promoting stakeholder engagement.

## Introduction

The European Union’s (EU) Green Deal emphasizes the need for innovative techniques, such as new genomic techniques (NGTs), to enhance the sustainability of food systems^1,2^. Unlike previous genetic modification techniques, NGTs allow for smaller and more targeted modifications without introducing foreign DNA to the target plants, potentially leading to crops with increased yield, improved nutritional characteristics, and greater resilience to abiotic and biotic stressors. Meanwhile, the EU food system faces substantial challenges in managing invasive alien species (IAS) and safeguarding plant health^3^. In this respect, NGTs offer a promising sustainable solution by enhancing the efficiency of plant breeding and enabling the precise introduction of specific beneficial traits to optimise the performance of elite crop germplasm^4,5^. If conventional breeding has generated elite germplasm that performs well for ten different traits but poorly on one specific trait (e.g., susceptibility to a specific disease), then NGTs can fix that last trait to perfect the cultivars. Elite crop germplasm refers to highly developed, superior genetic material that has undergone extensive selection, breeding, and optimization for specific traits, and includes commercial varieties and advanced breeding material.

The European Court of Justice ruling (Case C-528/16) in July 2018 classified plants derived from mutagenesis as genetically modified organisms (GMOs), exempting only those with a long history of safe use from regulation^6,7^. The approval process for GMOs, established in 2001, has slowed plant breeding innovations due to lengthy risk assessments and political decisions, resulting in longer approval times than in the U.S. and China and a lower number of approved events^8,9^. However, a 2021 European Commission (EC) study highlighted the potential of NGTs to contribute to sustainable agri-food systems aligned with the European Green Deal^10^, and alternative options for regulating NGT-derived plants in the EU are legally feasible and scientifically justifiable^11^. Brookes^12,13^ found that genetically modified crops reduced the environmental impact of insecticide and herbicide use by 17.3 per cent. They also significantly reduced greenhouse gas emissions by using reduced or no-till systems. However, Noack et al.’s^14^ review showed mixed effects of GM crop adoption on biodiversity, deforestation, and human health, with effects varying by GM trait and geographic scale. In a meta-study, Klümper and Qaim^15^ found that, on average, GM technology has reduced chemical pesticide use by 37%, increased crop yields by 22%, and increased farmers’ profits by 68%. Impacts vary, especially by modified crop trait and geographic region. Gusta et al.^16^ and Smyth et al.^17,18^ show that the adoption of herbicide-resistant canola has changed weed control practices in Canada, leading to shifts from soil-incorporated to foliar-applied post-emergent herbicides. As a result, the environmental impact of canola production, based on a modified Environmental Impact Quotient, dropped by 59% between 1995 and 2006.

In 2023, the Commission proposed a new legislative framework for plants obtained through certain NGTs (hereinafter referred to as the NGT proposal)^19^. In December 2025, the European Parliament, Council, and Commission reached a provisional political agreement on this new regulation. The proposal prompted varied reactions from agricultural organizations, civil society groups, and scientific bodies due to its broad implications for plant breeding and agricultural innovations^20^. The opposing stances of stakeholders towards NGTs are similar to those taken towards other biotechnologies. They are rooted in the perceived risks of NGTs, which range from basic biology to socio-economic factors^21^, but are often also strongly influenced by lobby groups^22^. Foodwatch criticized the proposal as a major setback for consumer rights, stating that removing mandatory labeling and risk assessment for Category 1 NGT plants would deny consumers transparency about their food and benefit large biotech companies, while the claimed sustainability benefits were seen as unproven assertions rather than evidence-based policy^23^. Similarly, IFOAM Organics Europe warned that changing the regulation of NGTs could undermine biosafety, reduce consumer transparency, threaten organic farming, and concentrate seed patents among a few corporations^24^. In contrast, EuropaBio saw it as a positive first step toward modernizing the EU’s GMO framework, urging broader regulatory reforms^25^, and the German National Academy of Sciences Leopoldina (with the DFG) supported the proposal as a scientifically sound framework that could enhance the sustainability and competitiveness of European agriculture^26^.

The sustainable protection of plant health from harmful pests and diseases is seen as crucial for the future of the agricultural sector and is dependent on the development of new technologies and innovations. The outlook for emerging technologies in the agri-food sector emphasizes the need for an environment that balances innovation with safety, environmental stewardship, and market fairness. By fostering an environment that encourages innovation while prioritizing safety and sustainability, the EU can aim to keep its agricultural sector competitive and resilient over the long term. This approach is intended to enable the sector to address the evolving demands of society and the environment and affect various EU policies such as the Zero Pollution Action Plan and the Farm to Fork Strategy^2,27,28^.

The control of IAS has become increasingly important due to their global rise over the past 200 years and ongoing threats to human health, food security, biodiversity, agriculture, forestry, and the economy^29–33^. Beyond direct damage, IAS incur substantial costs for prevention, control, and management^34^. The EU has taken substantial steps to combat biological invasions through the IAS Regulation^35^ and the Plant Health Law^36^, which aim to prevent IAS introduction and spread, ensure safe trade, and mitigate climate change impacts on crops and forests. The IAS Regulation emphasizes prevention as the most cost-effective approach, followed by early detection and rapid eradication. However, achieving complete prevention is often impractical, and developing an effective early detection system remains a challenge^37^.

Currently, the primary preventive strategy against pests and diseases at the farm level is the ex-ante application of pesticides^38^. However, the extensive use of chemical pesticides raises societal concerns due to risks to human health, the environment, and biodiversity^39–41^. The 1107/EC2009 Directive on the sustainable use of pesticides stressed reducing the use and risk of hazardous pesticides and investing in naturally derived low-risk pesticides (LRPs) as alternatives^42^. LRPs entail pest control products formulated with active substances that pose minimal hazard to human health and the environment. Examples of different LRP categories include biologicals, natural substances (e.g., plant extracts), semiochemicals, and double-stranded RNAs triggering RNA interference^43^. LRPs are gaining ground in the global pesticide market as substitutes for chemical pesticides^44,45^, but their overall share is still relatively small, estimated at 5% to 8% of the global market (Bullion and Shoham, 2022). In May 2020, the EC proposed a 50% reduction in the use and risk of chemical pesticides by 2030 as part of the Farm to Fork Strategy^2^. However, in 2024, the EP rejected this proposal after protests from farmers across the EU (EP, 2023).

Farmers need effective and economically viable pesticide alternatives that, at the same time, confer lower environmental and human health risks^46^. If solutions do not ensure that crop yields are not reduced and food prices do not increase, this could affect global agricultural trade markets and jeopardize global food security^47^. A promising solution in this respect is using NGTs to create pest-resistant crop cultivars and LRPs^19,48^.

## EU NGT proposal

### Overview of the NGT proposal

**Fig. 1:**
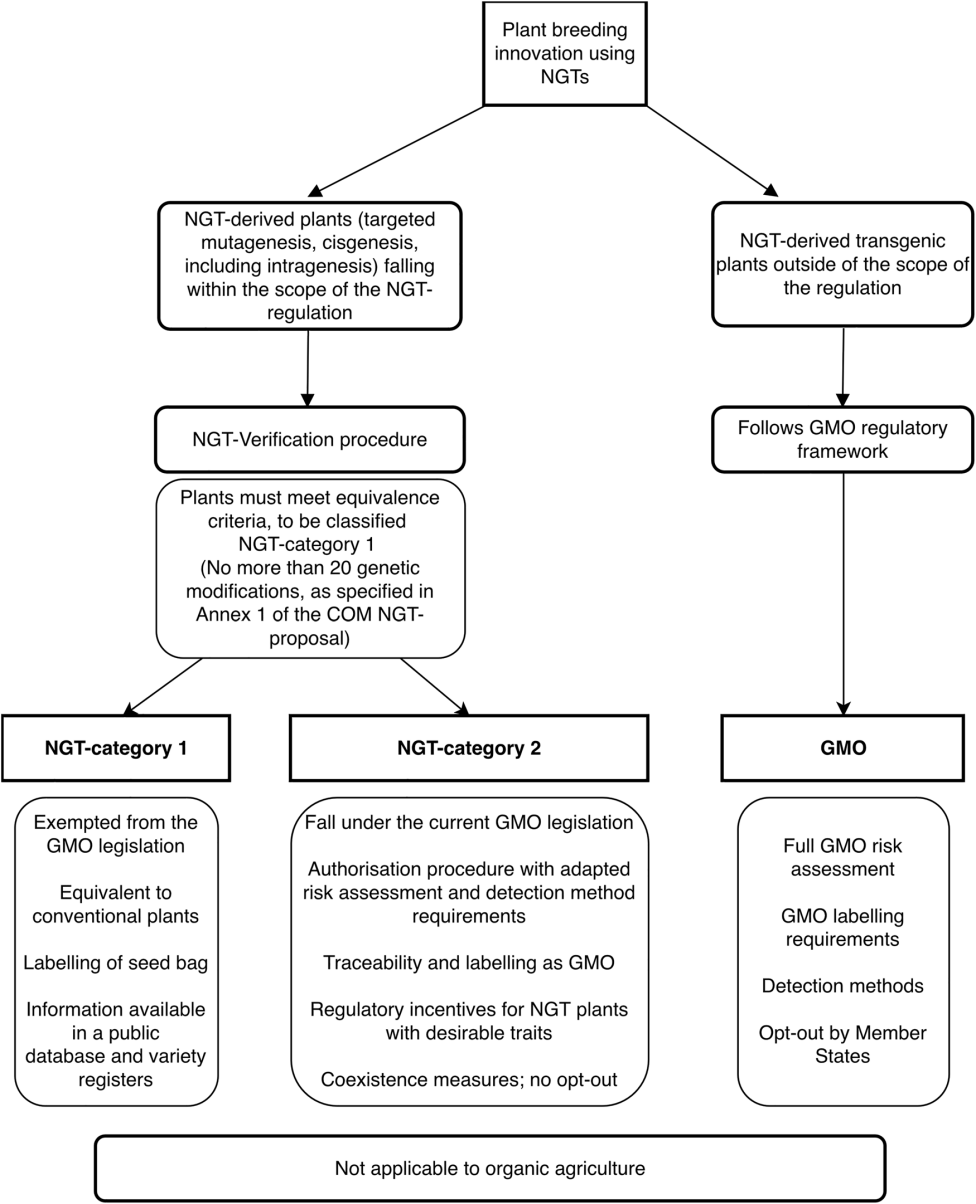
Schematic overview of the EC's proposal for regulating plants produced by NGTs.

The EC published the NGT proposal in July 2023, covering targeted mutagenesis and cisgenesis (incl. intragenesis), while transgenic plants will remain subject to GMO legislation. All NGT plants will continue to be classified as GMOs, but their regulatory requirements will change. Figure 1 shows that this proposal foresees simplifying the current authorization procedure for NGT-derived plants. It introduces two categories of NGT plants: firstly, “category 1 NGT plants”, which are produced via targeted mutagenesis or cisgenesis and might occur naturally or through conventional breeding techniques. After passing a verification procedure, NGT plants that align with the equivalence criteria will fall into category 1 and be exempted from the GMO legislation and its requirements. Secondly, NGT plants that do not meet the equivalence criteria would fall into “Category 2 NGT plants”. These will still follow current GMO legislation, but risk assessment, detection methods, and monitoring will be tailored to specific risk profiles due to their non-conventional genomic modifications. The scope of the environmental risk assessment is limited and may not require a monitoring plan^49^.

The deliberate release and placing on the market of NGT plants would, therefore, be subject to one of two procedures: verification for “Category 1 NGT plants” to establish equivalence with conventional products and authorization for “Category 2 NGT plants”. However, “Category 1 NGT plants” are treated as regulated GMOs in relation to organic production. For “Category 2 NGT plants,” EU countries must implement coexistence measures to prevent the unintentional presence of such NGT plants in other products. The possibility for EU countries to restrict or prohibit the cultivation of GMOs under Directive 2001/18 does not extend to such NGT plants.

### NGT proposal’s impact on the R&D and market adoption of NGT plants

The NGT proposal is expected to decrease the time and financial resources needed for an applicant to get an NGT plant cultivar placed on the market. This decrease could lead to a reduction of the minimum market area needed for developing new plant traits, making innovation more economically viable^50^. The effect will be most significant for NGT1 applications, as the verification process is expected to be quicker than the current GMO authorization. NGT2 plants are also anticipated to require fewer resources for authorization under the revised risk assessment. Overall, the likelihood that a product can be admitted to the market after the R&D process is expected to increase for all applications due to a more differentiated and non-restrictive regulatory approach.

An important consideration regarding the impact of the NGT proposal is whether plants and food products derived from the NGT1 category need to be labeled and whether traceability and coexistence measures will apply. According to the latest provisional trilogue agreement (December 2025), NGT1 food and feed products will not be labeled, while seeds and reproductive material will be labelled. Implementing labelling, traceability, and coexistence policies incurs additional costs at various stages, including R&D, production, processing, and retail^51^. Wesseler et al.^52^ demonstrated that requiring compliance with coexistence regulations for crops derived from NGTs could decrease welfare in the EU; the economic impacts vary substantially depending on the specific labeling policy adopted.

The magnitude of the NGT proposal’s impact will also depend on the share of applications falling under Categories 1 or 2. Bohle et al.^53^ indicate that 94% of the affected NGT plants would be classified as NGT1 and follow the streamlined verification procedure, which uses fewer resources^54^. This increase in efficacy is expected to increase companies’ willingness to invest in developing new NGT plants, leading to an increase in private R&D^55^.

The traits developed for NGT plants will affect whether the NGT proposal will have more economic, environmental, or social impact. R&D on NGT-derived plants is well underway, with the USA, China, and the EU publishing the most research on NGT plants. The majority of traits that have been developed until 2022 fall into three categories: First, improved plant quality for food and feed production; second, improved agronomic traits related to plant yield and growth to increase productivity or prevent pre-harvest losses; third, improved disease resistance to reduce the need for crop protection products in agricultural production^56^. Due to the variety of traits in development, NGTs could help to introduce a greater diversity of crops to organic agriculture, thereby increasing its efficiency and resilience^57^.

## Impact on agriculture

### Key emerging technologies and innovations in agriculture, food, and biomass production

The objectives of the European Green Deal are closely linked to transforming the agri-food sector, which depends on the successful adoption of emerging technologies. There are 62 key technologies and innovations in agriculture, food, and biomass production, which were identified by a comprehensive methodological approach that combines artificial intelligence-based technology assessments, systematic literature reviews, and qualitative comparative analyses. Technology and innovation clusters include one focused on emerging technologies that modify organisms’ genes to create new products. Other clusters involve integrative technologies designed to decentralize the existing food system and improve access to nutritious food and information. Additionally, some technologies use sensors and robots in agricultural production, while disruptive digital technologies, such as DNA fingerprinting for food, enhance the transparency and resilience of food systems^58^.

Genome editing, biofortification, and in vitro cultivation of plants, all related to genomic techniques, hold great promise for advancing agricultural sustainability. These innovations have the potential to enhance crop resilience, improve nutritional content, reduce dependency on chemical fertilizers, and increase overall agricultural productivity. By leveraging precise genetic modifications, bioengineered crops can better withstand environmental stresses. Furthermore, biofortification addresses micronutrient deficiencies in staple foods. Additionally, nitrogen-fixing bacteria provide a sustainable alternative to chemical nitrogen fertilizers, reducing their environmental impact, while in vitro culture techniques enable the rapid propagation of improved plant varieties and the elimination of viral infections in propagation materials. These approaches could be crucial in creating a more sustainable and resilient agricultural system.

### NGT proposal’s impact on the role of NGTs among these technologies and innovations

Innovations in food biotechnology are continuously transforming global food production and plant breeding. These advances in biotechnology include NGTs that enable precise changes in the genome and retard unintended effects while they improve crop resilience and increase the efficiency of desired traits^59^. The NGT proposal considerably impacts technologies and innovations in the agri-food sector. According to Purnhagen et al.^11^, a change in EU regulation on NGTs enables increased agricultural innovation and sustainability in the region and promotes international trade.

One significant impact of NGTs, along with other emerging technologies and innovations, relates to their environmental effects. Research shows that NGTs can reduce the resources used in farming^60^. However, the anticipated benefits of NGTs do not always materialize; for instance, a farmer may continue to water plants excessively, even when an NGT variety requires less water. Additionally, some studies suggest that the environmental issues linked to the release of NGT organisms could be as lasting as those caused by pesticides^61,62^. While NGTs present numerous advantages, it is crucial to approach their use with caution and effective management to avoid long-term ecological damage, similar to the current environmental challenges posed by pesticide application. These potential concerns underscore the importance of assessing how the NGT proposal influences the adoption, regulation, and integration of NGTs within the wider context of new agricultural technologies and innovations.

## Impact on LRPs and IAS management

### Regulatory constraints hampering the full market potential of LRPs

LRPs still do not realize their full market potential due to regulatory constraints, beginning with ambiguity in terminology. The designation of “low-risk” is granted to an active substance only after this active substance has met specific data requirements and risk assessment criteria. However, this terminology does not allow for an earlier identification of active substances that can potentially be considered “low-risk”. For this purpose, the term potential LRP is used to refer to substances that have the potential to be recognized as “low-risk”^43^. This aligns with the term “active substance of low concern” introduced in a tender call (GP/EFSA/PLANTS/2023/04) of the European Food Safety Authority (EFSA). When an active substance is approved as low-risk, it benefits from an extended approval period of 15 years compared to the standard 10, and data protection is increased from 10 to 13 years. Moreover, low-risk status can be used in advertising to enhance marketability.

Regardless of the advantages of pLRPs, within the EU, the current approval process for the respective active substance follows the same regulatory approach and data requirements as for chemical pesticides(except micro-organisms)^63^. Note that, within this context, the term “chemical pesticides” is used to refer to the specific plant protection products targeted under the Farm to Fork Strategy goals, i.e., LRPs of chemical origin are excluded from this designation This undifferentiated approach can result in an unnecessary regulatory burden^64^ and the absence of specific regulatory risk assessment procedures has been recognized as a major barrier to introducing pLRPs in the EU market. Balog et al.^3^ concluded that procedural times in the EU are longer than in the USA, causing the EU to lag behind other major agricultural markets regarding LRPs for farmers. The economic costs of waiting for approval, along with R&D expenses, increase costs for companies and hinder investment. Additionally, the prolonged approval process limits benefits from pLRPs, resulting in opportunity costs for farmers and consumers in the EU^6^.

### NGT proposal’s Impact on LRPs and IAS management

The proposed regulation on plants obtained by NGTs and their food and feed could impact the regulatory constraints affecting the market reach of pLRPs. While some studies suggest that further adoption of genetically modified crops could benefit the environment and human health by reducing pesticide use^65^, others argue that the environmental problems caused by the interaction of several NGT organisms and the environment may be as persistent as those caused by pesticides^61^. Koller et al. reported that potential risks include disruption to the plant microbiome and pollinator interactions, reduced resilience to biotic and abiotic stressors, evolutionary mismatches that affect future adaptation, and threats to biodiversity arising from the uncontrolled spread of NGT organisms and their offspring. Considering the potential global reduction in pesticide use due to higher adoption of genetically modified crops^15^, traits associated with higher pest resistance may reduce the overall need for pesticides, both from chemical and low-risk origins.

Additionally, achieving the targets of the Green Deal needs to go beyond the ban on single active substances^46^. It is essential to fortify farmers’ IAS management toolkit with innovative alternatives for crop protection, such as biocontrol agents, precision agriculture, biostimulants, LRPs, and resistant cultivars through breeding^38^. Reducing the number of active ingredients poses a high risk of pest resistance development, raising the costs of effective pest management. The role of biotechnology in overcoming these challenges has been recognized^19^. Therefore, removing legislative barriers to new breeding techniques, such as NGTs, is crucial to minimizing the time needed for the introduction of resistant varieties and to contributing to farmers’ income by decreasing pesticide application^38^. Reducing the time needed to translate R&D into products available to farmers in the EU can increase the competitiveness of EU agriculture and farm income^66^.

### Broadening the sustainability lens: environmental, economic and human health dimensions

Adopting NGTs in the EU under the NGT proposal could have a significant impact on sustainability, extending beyond their effects on pests and diseases. The previously cited meta-analysis by Klümper and Qaim^15^ suggests lower environmental pressure from agrochemicals and a smaller ecological footprint for crop production with the wider use of NGTs. Furthermore, long-term analyses suggest that reducing reliance on pesticides through genetically modified crops can substantially decrease the environmental impact quotient of crop protection practices, thereby mitigating the impact on agroecosystems, such as exposure of non-target species^13^.

Genetically modified and gene-edited crops can contribute to lower input costs and greater profitability for producers by making pest and weed management more efficient, reducing reliance on agrochemicals, and protecting against crop losses. Institutional assessments have found that farmers who adopt genetically improved crops experience higher yields and reduced production costs, primarily due to more effective control of target pests and weeds, and associated reductions in chemical use^67^. These economic gains extend beyond individual farms. Increased productivity and reduced input requirements can support food security objectives, particularly in regions where resources for conventional chemical inputs or mechanised control are limited. Enhancements in stress tolerance and disease resistance, which are becoming more accessible through genome editing, could further increase economic resilience to climate variability^68^. Jin et al.^66^ found that the cost of postponing adoption of NGTs ranges from losses of −$1.1 billion in Brazil to gains of $18.5 billion in Europe.

Reducing the need for chemical pesticides and herbicides has direct and indirect benefits for human health. Studies of the impact of GM crops report substantial reductions in pesticide poisonings among farmers and agricultural workers where insect-resistant crops have replaced frequent spraying. Similarly, GM crops that are more resistant to insect damage have been linked to lower mycotoxin levels in harvested grain, which could reduce the risk of carcinogens entering the food supply^69^. NGTs also have the potential to increase food security by providing higher levels of micronutrients and calories, which are important for improving child health^70^. The EU-SAGE^71^ database lists more than a hundred examples of NGT applications in this regard.

## Conclusion

The proposed EU regulation on NGTs marks a significant change in the legislative landscape, with important implications for the agricultural sector and the overarching goals of the Green Deal. By streamlining the approval process for plants derived from NGTs, the regulation aims to promote innovation and sustainability in plant breeding, potentially resulting in more resilient and productive crops. This regulatory shift is expected to reduce the time and resources needed for market approval, particularly for “Category 1 NGT plants.” As a result, it is likely to encourage private-sector investment in agricultural research and development.

The NGT proposal is in line with the European Green Deal and its related strategies, including the Farm to Fork and Biodiversity Strategies. NGT Plants have the potential to reduce pesticide use and associated risks. The Farm to Fork Strategy aims to transition to a sustainable food system that has either a neutral or positive environmental impact, mitigates climate change, and reverses biodiversity loss, all while ensuring food security, nutrition, and public health. A study by the Joint Research Centre identified 409 early and advanced research and development applications of NGT. These applications include enhancing tolerance to biotic and abiotic stress, modifying plant composition, improving yield, and enhancing storage performance^72^.

The impact of NGTs extends beyond plant breeding to encompass broader agricultural practices, including pest and IAS management. Developing pest-resistant NGT plants could contribute to the EU’s goals of reducing pesticide use and associated risks, aligning with the objectives of the Green Deal. However, the regulation’s success depends on its implementation, particularly regarding labeling, traceability, and coexistence measures, which could impose additional costs and affect market dynamics.

NGTs could help reduce European farmers’ reliance on pesticides to tackle pests and diseases, but the existing regulatory framework for pLRPs poses a barrier to their full market potential. By streamlining the approval processes for NGTs and pLRPs, the development of diverse products for farmers could be increased. This increase would promote the adoption of sustainable agricultural practices, ensuring farmers have access to practical and economically viable alternatives to chemical pesticides.

In conclusion, the EU’s NGT regulation presents an opportunity to advance agricultural innovation and sustainability. However, achieving these goals requires a balanced approach that addresses regulatory challenges, promotes stakeholder engagement, and ensures that the benefits of NGTs and pLRPs are fully realized. By fostering a flourishing environment for technological advancements, the EU can support a resilient and sustainable agricultural sector that meets the evolving demands of society and the environment.

## Data Availability

No datasets were generated or analysed during the current study.
